# Supplementary material for: Magnitude of ectopic pregnancy, management methods, and its associated factors among pregnant women attending Ambo University Referral Hospital in Oromia Regional State, Ethiopia: A seven years retrospective institutional based cross-sectional study
Source: PLOS Glob Public Health. 2025 Jun 5;5(6):e0004611. doi: 10.1371/journal.pgph.0004611 (PMC12140202; doi:10.1371/journal.pgph.0004611)
Supplement: S1 Checklist — (DOCX) [file pgph.0004611.s001.docx]

**English version checklist.**

**Part I Socio-demographic characteristics**

| **Code** | **Variable** | **Response** | **Remark** |
| --- | --- | --- | --- |
| **101** | Age | ---------------in years |  |
| **102** | Residence | 1. Rural 2. Urban |  |
| **103** | Marital status | 1. Married 2. . Widowed 3. Divorced 4. single |  |
| **104** | Occupation | 1. Housewife  2. Government employee  3. Merchant  4. Daily laborer |  |
| **105** | Educational level | 1. Unable to read and write  2. 1-8th grade  3. 9-12th grade  4. College or University |  |
| **106** | Religion | 1. Orthodox  2. Muslim  3. Protestant  4. Other/ specify |  |
| **107** | Ethnicity | 1. Oromo  2. Amhara  3. Tigray  4. Guraghe  5. Other/ specify------------ |  |

**Part II Clinical presentation and Diagnosis of Ectopic pregnancy**

| 108 | What is the chief complaint of the patient? | 1. Abdominal pain. 2. Vaginal bleeding. 3. Amenorrhea. 4. Syncope. |  |
| --- | --- | --- | --- |
| 109 | What is the type of ectopic pregnancy? | 1. Ruptured 2. Unruptured |  |
| 110 | What is the gestational week? | 1. < 7 week 2. 7-9 week 3. > 9 week |  |
| 111 | How Ectopic pregnancy was diagnosed? | 1. Clinical only 2. U/S only 3. Clinical and U/S 4. Clinical and culdocentesis 5. Clinical, culdocentesis and U/S 6. Intraoperative |  |
| 112 | Urine HCG test result | 1. Negative 2. Positive |  |
| 113 | Hemoglobin level of the patients? | 1. < 5g/dl 2. 5g/dl- 10g/dl 3. >10g/dl |  |
| 114 | Estimated blood lost during the intraoperative process? | 1. <500ml 2. 500ml -1000ml 3. 1000ml -1500ml 4. >1500ml |  |
| 115 | The blood group of the patient | 1. ­­­­­­­­­­­­­­­­­­A 2. _B_ 3. _O_ 4. _Others_ |  |

**Part III past Obstetrics and surgical History.**

| **No.** | **Variable** | **Response** | **Remark** |
| --- | --- | --- | --- |
| **116** | State of pregnancy | 1. Intrauterine pregnancy  2. Ectopic pregnancy |  |
| **117** | If Qno. 9 is Ectopic pregnancy where is the site of pregnancy? | 1. Tubal  2. Abdominal  3. Ovarian  4. Cervix  5. Other Specify--------------- |  |
| **118** | If Qno10 is tubal where is the site? | 1. ampulla  2. isthmus  3. Fimbrie |  |
| **119** | Do you have a Previous history of Ectopic pregnancy? | 1. Yes  2. No |  |
| **120** | Previous history of abortion? | 1. Yes  2. No |  |
| **121** | If Qno.13 is yes what is the type of abortion? | 1. Spontaneous only  2. Induced only  3. Both |  |
| **122** | If Qno.14 is both how many times spontaneous abortion occurs? | 1. 1 2. 2 and above |  |
| **123** | If Qno. 14 is both how many times induced abortion occurs? | 1. 1 2. 2 and above |  |
| **124** | Previous history of appendectomy | 1. Yes  2. No |  |
| **125** | Previous history of tubal surgery | 1. Yes  2. No |  |
| **126** | Previous history of cesarean section | 1. Yes  2. No |  |
| **127** | Parity | 1. 0  2. 1  2. 2  3. 3  4. 4  5. 5 and above |  |
| **128** | Previous history of recurrent STI/STD | 1. Yes  2. No |  |
| **129** | Previous history of PID |  |  |
| **130** | History of infertility | 1. Yes  2. No |  |
| **131** | History of IUCD use | 1. Yes  2. No |  |
| **132** | History of OCP use | 1. Yes  2. No |  |
| **133** | History of injectable contraceptive use | 1. Yes  2. No |  |
| **134** | History of implant |  |  |

**Part III Behavioral History**

| **No.** | **Variable** | **Response** | **Remark** |
| --- | --- | --- | --- |
| **135** | Cigarettes smoking | 1. non-smoker  2. Occasional smokers  3. regular smokers |  |
| **136** | If Yes to 24 how long ago did you start smoking? | **----------------** |  |
| **137** | If yes to 24, how often were you smoking? | 1. Daily  2. Once a week  3. 3 times per week  4. Once a month |  |
| **138** | The number of cigarettes used? | **----------------** |  |
| **139** | A history of alcohol drinking? | 1. Yes  2. No |  |
| **140** | If Yes to Q28 How often do you have a drink containing alcohol? | 1. Less than monthly  3. 2-4 times a month  3. 2-4times a week  4. 4/more times a week |  |
| **141** | Outcome of ectopic pregnancy? | 1. Favorable 2. Unfavorable |  |
