# Supplementary material for: Magnitude of ectopic pregnancy, management methods, and its associated factors among pregnant women attending Ambo University Referral Hospital in Oromia Regional State, Ethiopia: A seven years retrospective institutional based cross-sectional study
Source: PLOS Glob Public Health. 2025 Jun 5;5(6):e0004611. doi: 10.1371/journal.pgph.0004611 (PMC12140202; doi:10.1371/journal.pgph.0004611)
Supplement: Attachment — Submitted filename: Response to Reviewers3.docx [file pgph.0004611.s004.docx]

**Response to Reviewers**

**Dear Editor,**

Submission ID PGPH-D-24-03013

We would like to sincerely thank each and every one of the reviewers of our work. We really appreciate all of the reviewers' time and work in assessing our article, and we think your knowledge tremendously improves its quality and results in a stronger revised version.

Thank you once again for giving us the opportunity to revise and resubmit this manuscript. We appreciate the time you invest to review this manuscript and has incorporated the suggested changes into the manuscript to the best of our ability. The manuscript has certainly benefited from these insightful revision suggestions. Please consider the revised version of our article “**MAGNITUDE OF ECTOPIC PREGNANCY, MANAGEMENT METHODS, AND ITS ASSOCIATED FACTORS AMONG PREGNANT WOMEN ATTENDING AMBO UNIVERSITY REFERRAL HOSPITAL IN OROMIA REGIONAL STATE, ETHIOPIA: A SEVEN YEARS RETROSPECTIVE INSTITIONAL BASED CROSS-SECTIONAL STUDY**” to be published in the “**PLOS Global Public Health ”**. The following pages are our point to point responses to the comments and suggestions raised by the editor. We hope that the revisions and accompanying responses will be sufficient to make the manuscript suitable for publication in **PLOS Global Public Health.**

Yours sincerely.

Nigussie Tesfaye Gizaw

**Dear Reviewer**

1. Please send a completed 'Competing Interests' statement, including any COIs declared by your co-authors. If you have no competing interests to declare, please state "The authors have declared that no competing interests exist".

Answer. Thank you very much for your thoughtful and constructive comments you provided on our manuscript. Yes it is corrected in the manuscript as you provided your feedback.

2. Please amend your detailed Financial Disclosure statement. This is published with the article. It must therefore be completed in full sentences and contain the exact wording you wish to be published

Answer. I sincerely appreciate your recommendations and insightful comments. Yes it is corrected in the manuscript as you provided your manuscript.

3. In the online submission form, you indicated that "Upon a reasonable request, the corresponding author will provide the tool used for data collection and dataset analysis in this study.".

All PLOS journals now require all data underlying the findings described in their manuscript to be freely available to other researchers, either

1. In a public repository,

2. Within the manuscript itself, or

3. Uploaded as supplementary information.

Answer. Thank you very much, your insightful and helpful feedback on our manuscript is greatly appreciated.

All relevant data are within the manuscript and its Supporting information files.

4. Please upload all main figures as separate Figure files in .tif or .eps format only and remove the embedded figures from the manuscript.

Answer. We sincerely appreciate your thoughtful and beneficial comments on our manuscript.

Yes we tried to correct and upload as supporting information.
