# Supplementary material for: Magnitude of ectopic pregnancy, management methods, and its associated factors among pregnant women attending Ambo University Referral Hospital in Oromia Regional State, Ethiopia: A seven years retrospective institutional based cross-sectional study
Source: PLOS Glob Public Health. 2025 Jun 5;5(6):e0004611. doi: 10.1371/journal.pgph.0004611 (PMC12140202; doi:10.1371/journal.pgph.0004611)
Supplement: Attachment — Submitted filename: Response to Reviewers4.docx [file pgph.0004611.s005.docx]

**Response to Reviewers**

**Dear Editor,**

Submission ID PGPH-D-24-03013

We would like to sincerely thank each and every one of the reviewers of our work. We really appreciate all of the reviewers' time and work in assessing our article, and we think your knowledge tremendously improves its quality and results in a stronger revised version.

Thank you once again for giving us the opportunity to revise and resubmit this manuscript. We appreciate the time you invest to review this manuscript and has incorporated the suggested changes into the manuscript to the best of our ability. The manuscript has certainly benefited from these insightful revision suggestions. Please consider the revised version of our article “**MAGNITUDE OF ECTOPIC PREGNANCY, MANAGEMENT METHODS, AND ITS ASSOCIATED FACTORS AMONG PREGNANT WOMEN ATTENDING AMBO UNIVERSITY REFERRAL HOSPITAL IN OROMIA REGIONAL STATE, ETHIOPIA: A SEVEN YEARS RETROSPECTIVE INSTITIONAL BASED CROSS-SECTIONAL STUDY**” to be published in the “**PLOS Global Public Health ”**. The following pages are our point to point responses to the comments and suggestions raised by the editor. We hope that the revisions and accompanying responses will be sufficient to make the manuscript suitable for publication in **PLOS Global Public Health.**

Yours sincerely.

March 23, 2025

Nigussie Tesfaye Gizaw

**Dear Reviewer**

1. Reviewer #1: The authors present a very detailed and thorough evaluation of ectopic pregnancies that were diagnosed and treated at a referral hospital during a period of 7 years. Although the study was conducted properly and the manuscript is well written, several corrections should be made to achieve better comprehension. First, the introduction part should be condensed into one page. Second, the authors should mention about the factors that limit the power of their study (i.e. retrospective design, recall/recording bias). Third, the authors should replace the references that were published before 2010 with newer and more up-to-date ones if possible.

Answer. Thank you very much for your thoughtful and constructive comments you provided on our manuscript. Yes it is corrected in the manuscript as you provided your feedback. The introduction also condensed in one page, the weakness of retrospective study also mentioned and the reference citation were also updated.

Reviewer #2: Dear Authors, Thank you to allow me to review this interesting manuscript. It is a retrospective analysis on the ectopic pregnancies treated in Ambo University Referral Hospital in Oromia Regional State, Ethiopia. The authors have performed a deep analysis on the risk factors (social and demographics) the type of symptoms, diagnosis and therapies. Also the statistical analysis was correctly performed. The figures are clear. The sample is very large. However i have some questions:

- Why some patients underwent oophorectomy? They had an ovarian ectopic pregnancy? This should be specified

To be more readable I suggest to shorten the discussion section. Furthermore, the manuscript report only surgical treatment and this is justified by the economic and social context. However author correctly report information about the medical treatment. They report complications related to the drug side effects but they aren't the only type of complications. Sometimes are reported also organ damage or other types of complications related to the ectopic pregnancy itself even when this has resolved with medical therapy. I suggest to read and cite 10.3390/jcm12237396.

Answer. I sincerely appreciate your recommendations and insightful comments. The question regarding other complications of medical treatment were added in the manuscript.

The surgical treatment underwent for many reasons: conditions of the patients, when medical treatment is not an option, for successful treatment, to remove the ectopic tissue and other social and economic context matters.

Reason for oophorectomy: nine cases of ovarian ectopic pregnancy were recorded.
